# Herd-level animal management factors associated with the occurrence of bovine neonatal pancytopenia in calves in a multi-country study

**DOI:** 10.1371/journal.pone.0179878

**Published:** 2017-07-05

**Authors:** Carola Sauter-Louis, Bryony A. Jones, Jörg Henning, Alexander Stoll, Mirjam Nielen, Gerdien Van Schaik, Anja Smolenaars, Matthijs Schouten, Ingrid den Uijl, Christine Fourichon, Raphael Guatteo, Aurélien Madouasse, Simon Nusinovici, Piet Deprez, Sarne De Vliegher, Jozef Laureyns, Richard Booth, Jacqueline M. Cardwell, Dirk U. Pfeiffer

**Affiliations:** 1 Clinic for Ruminants with Ambulatory and Herd Health Services at the Centre for Clinical Veterinary Medicine, Ludwig-Maximilians-University Munich, Oberschleissheim, Germany; 2 Veterinary Epidemiology, Economics & Public Health Group, Royal Veterinary College, Hawkshead Lane, North Mymms, Herts, United Kingodm; 3 Institute of Epidemiology, Friedrich-Loeffler-Institute, Suedufer 10, Greifswald–Isle of Riems, Germany; 4 School of Veterinary Science, The University of Queensland, Gatton, Australia; 5 Department of Farm Animal Health, Faculty of Veterinary Medicine, University of Utrecht, Yalelaan 7, CL Utrecht, The Netherlands; 6 GD Animal Health, Arnsbergstraat 7, AA Deventer, The Netherlands; 7 INRA, UMRI300 Biology, Epidemiology and Risk Analysis in Animal Health, INRA, LUNAM Université Oniris, Ecole nationale vétérinarie, agroalimentaire et de l’alimentation Nantes Atlantique, Atlanpole La Chantrerie, BP Nantes, France; 8 Department of Internal Medicine and Clinical Biology of Large Animals, Faculty of Veterinary Medicine, Ghent University, Salisburylaan 133, Merelbeke, Belgium; 9 Department of Reproduction, Obstetrics and Herd Health, Faculty of Veterinary Medicine, Ghent University, Salisburylaan 133, Merelbeke, Belgium; 10 Department of Pathology and Infectious Diseases, Royal Veterinary College, Hawkshead Lane, North Mymms, Herts, United Kingdom; 11 School of Veterinary Medicine, City University of Hong Kong, Hong Kong, Hong Kong SAR; Atlantic Veterinary College, CANADA

## Abstract

Since 2007, mortality associated with a previously unreported haemorrhagic disease has been observed in young calves in several European countries. The syndrome, which has been named ‘bovine neonatal pancytopenia’ (BNP), is characterised by thrombocytopenia, leukocytopenia and a panmyelophthisis. A herd-level case-control study was conducted in four BNP affected countries (Belgium, France, Germany and the Netherlands) to identify herd management risk factors for BNP occurrence. Data were collected using structured face-to-face and telephone interviews of farm managers and their local veterinarians. In total, 363 case farms and 887 control farms were included in a matched multivariable conditional logistic regression analysis. Case-control status was strongly associated with the odds of herd level use of the vaccine PregSure® BVD (PregSure, Pfizer Animal Health) (matched adjusted odds ratio (OR) 107.2; 95% CI: 41.0–280.1). This was also the case for the practices of feeding calves colostrum from the calf’s own dam (OR 2.0; 95% CI: 1.1–3.4) or feeding pooled colostrum (OR 4.1; 95% CI: 1.9–8.8). Given that the study had relatively high statistical power and represented a variety of cattle production and husbandry systems, it can be concluded with some confidence that no other herd level management factors are competent causes for a sufficient cause of BNP occurrence on herd level. It is suggested that genetic characteristics of the dams and BNP calves should be the focus of further investigations aimed at identifying the currently missing component causes that together with PregSure vaccination and colostrum feeding represent a sufficient cause for occurrence of BNP in calves.

## Introduction

Until 2007, only sporadic cases of haemorrhagic disease in young calves had been observed. Starting that year, an increased number of young calves with haemorrhagic disease of unknown origin in dairy and beef herds were reported. The first cases of the syndrome, later named bovine neonatal pancytopenia (BNP), were reported in Bavaria in 2007, then in other parts of Germany and in other European countries [1] such as the Netherlands [2], Belgium [3], France [4], Great Britain [5], Italy [6], Ireland [7], Poland [8], Spain [9] and Hungary [10]. In 2011, the first cases of BNP were observed in New Zealand [11]. The total number of cases reported in all of the affected European countries (around 6000 cases until 2014) suggests that the disease is rare considering the large number of calf births [12].

Calves with BNP are aged 1 to 4 weeks when showing clinical signs of a haemorrhagic diathesis [13, 14]. Cases are characterised by leukocytopenia and thrombocytopenia, regardless of different thresholds used to define this disorder in different countries [13, 14]. The main pathological sign is a trilineage depletion of stem cells in the bone marrow (panmyelophthisis) in sternum or femur [13–15].

Early research into BNP examined different potential causes of haemorrhagic diathesis, such as various poisons and infections including BVDV (bovine virus diarrhoea virus), BTV (bluetongue virus), EHD (epizootic haemorrhagic disease), but diagnostic test results were all negative [13–16]. As the disease occurs in both sexes and in different breeds it was considered unlikely that a genetic factor would be the sufficient cause. However, a genetic predisposition was hypothesised, because of the sporadic pattern of the disease within herds [13].

Investigations of risk factors for BNP revealed an association between the development of BNP in calves and vaccination of their dams against BVD using PregSure® BVD (PregSure, Pfizer Animal Health) [3, 17–19]. However, these were relatively small-scale studies, and they therefore did not have sufficient statistical power to detect additional risk factors with small effect sizes. Other research into the aetiology of BNP suggested an autoimmune-mediated process, whereby alloantibodies are transferred from the cow to the calf via the colostrum. The involvement of colostrum as one mechanism for inducing the disease was demonstrated in two experimental feeding trials. In these studies, feeding colostrum from cows that previously had a calf with BNP to calves from other dams, which never had BNP-calves, resulted in BNP, suggesting an autoimmune-mediated process [1, 20].

Bridger et al. and Pardon et al. [21, 22] identified alloreactive antibodies in the sera of BNP-dams that bind to surface antigens on leucocytes of calves. Experimental immunisation of cows showed that different vaccination schemes have an effect on the level of antibody titres, increasing antibody titres to different degrees, and in turn affecting disease occurrence [23]. Although the association between vaccination of dams with PregSure and the occurrence of BNP was assumed early in the process, the small number of cases in relation to the high number of vaccinated cows suggested that other factors were contributing to the aetiology.

In a previously published study we explored animal-level risk factors for developing BNP [24]. Vaccination of the calf’s dam with PregSure was the only risk factor strongly associated with disease occurrence. It remains unknown what other factor(s) (or component causes) are required to constitute a sufficient cause for this disease, considering the widespread use of the vaccine (and feeding of colostrum from vaccinated dams) had still only resulted in a very low BNP incidence. Therefore, the aim of this case-control study was to identify herd-level management factors associated with the occurrence of BNP in a large sample of farms from four affected countries representing a variety of cattle production systems and husbandry conditions in Europe, including a variety of management factors which had not been included in previous studies, such as other vaccinations or routine treatments.

## Materials and methods

### Ethics statement

The research reported here does not involve data on human participants themselves, only on farm management practices they were using. All data were collected via questionnaires. All livestock owners were approached by their farm veterinarians, informed about the aim of the study and asked if they were willing to participate, thereby asking for their verbal consent. Questionnaires were only used with farmers who gave their verbal consent, which was noted on the questionnaire. Data being entered into the online database were anonymized by using the country code and the name of the veterinarian who provided the contact (in order to match the farms on veterinary practice). No names of farmers were recorded in the database. Livestock owners provided verbal consent for sampling their animals. Procedures on animals used in the calf-level study were in accordance with the ethical conditions for animal experimentation as mentioned in the European legislation (Directive 86/609/EEC). The blood samples collected from calves on the farms were taken for diagnostic purposes at the request of the owners as part of clinical veterinary practice. The samples were taken by the animal owners’ veterinarians; in the Netherlands and Belgium by project veterinarians on behalf of the owners’ veterinarians. Ethics approval for sampling of the animals in this study was not required for taking blood because this was done for diagnostic purposes by the animal owners’ veterinarians. This is consistent with Animal Welfare legislation in the four study countries (Belgium, France, Germany and the Netherlands).

### Study design

A case-control study was conducted that recruited BNP-affected farms (cases) from four countries: Belgium, France, Germany and the Netherlands—all countries with comparatively high BNP occurrence until mid-2010. The study was coordinated by staff from the Royal Veterinary College in the United Kingdom. Each case farm was matched to 2–4 control farms of the same production type and from the same veterinary practice to control for confounding.

A statistical power analysis was conducted to estimate the required sample size to detect odds ratios of less than 0.54 or higher than 1.6 with a power of at least 80%, assuming independence between observations and no matching, 10% risk factor prevalence in controls and a 95% confidence level for a 2-sided test. This resulted in a required sample size of 400 case herds and 1600 control herds across the project countries. Thus, the aim was to recruit approximately 100 case farms and 200–400 control farms in each country between 1st January and 31st December 2011. It was expected that the incidence of BNP cases during the data collection period would vary between the countries.

Additionally, a case-control study at animal level was conducted, investigating animal-level risk factors within BNP affected farms which has been published elsewhere [24]. The current study complements that previously published study by focussing on herd-level management factors.

### Definition of BNP case and matched control farms

A farm was classified as a case if it had at least one calf diagnosed with BNP in 2011. The case definition for a BNP calf was: aged ≤28 days with at least one of the following clinical signs of haemorrhage: multiple skin haemorrhages, melaena, petechiae in mucus membranes, sudden death, panmyelophthisis in bone marrow histology and/or thrombocytes below 150 x 109/litre and leucocytes <5 x 109/litre.

A farm was classified as a matched control if it was of the same production type and served by the same veterinary practice as the case farm and if it had never had a confirmed or suspected (by either the veterinarian or the farmer) BNP calf.

### Recruitment of farms

In each country veterinarians and farmers were encouraged to report suspected BNP cases to the project team. Recruitment of farms was adapted to the local situation. In Germany, a request to report suspected BNP cases together with a description of the study was published in the German Veterinary Association’s journal (“Deutsches Tierärzteblatt”) as well as in various local farming publications. In the Netherlands, BNP cases were reported by farmers and veterinarians as part of routine national surveillance. Additionally, the likelihood of reporting was further increased by announcements in the local farming press and veterinary journals. In Belgium, cases were also recruited as part of routine surveillance conducted by Ghent University and Animal Health Care Flanders (DGZ; Dierengezondheidszorg Vlaanderen), with farmers and veterinarians informed via a website and encouraged to report suspect calves. Furthermore, during the study several articles were published in farming journals to increase the awareness of farmers and thereby likelihood of reporting. In France, the study was advertised in the veterinary and farming press. For all countries, instructions and questionnaires were available in local language for download via a website, and veterinarians were able to contact the project team directly.

In each country, farms that reported suspected BNP cases were visited by a veterinarian (either a local or a project veterinarian) and whole blood samples were collected from calves suspected to be affected. In Germany, all blood samples were sent to the laboratory of the Ludwig Maximilian University (LMU) Munich Clinic for Ruminants. Carcasses of suspected cases were sent to the locally responsible government animal health centres within Germany and the post-mortem examination reports were sent to the project team at the LMU Munich. In the Netherlands, all blood samples and carcasses were sent to GD Animal Health in Deventer. In France, veterinarians involved in the on-farm data collection analysed the blood samples in their own laboratory if possible, or in a human diagnostic laboratory. Gross-pathological signs were recorded during the post-mortem examination by these veterinarians and bone-marrow samples stored in formaldehyde were sent to Oniris for histological analysis. In Belgium, blood samples from BNP suspected calves were examined in the laboratory of Ghent University, Department of Internal Diseases of Large Animals, and carcasses were sent to DGZ for necropsy. Post mortem reports were sent to the project office. A bone marrow sample was sent to the Department of Pathology at the Veterinary Faculty for final diagnosis of BNP.

If a BNP suspected calf was confirmed to have BNP based on the criteria described above, the farm was classified as a ‘case-farm’ and visited for a detailed interview. The approach to control farm selection varied by country. In Germany, the veterinarian reporting the BNP case was asked to provide the contact details of six of their clients who had never had a suspected or confirmed BNP calf on their farm. From these six farms, four were selected randomly. In the Netherlands, 10 herds of similar type and from the same veterinary practice were ranked by distance to the case herds and contacted by the investigators for willingness to participate in the study, starting with the first herd on the list and continuing until four control herds were recruited. In Belgium and France, the veterinarian for each case farm was asked to provide contact details of four of their clients with farms of the same management type.

### Data collection

The data for this study was collected by structured interviews using a questionnaire. Given the lack of knowledge of likely risk factors, apart from feeding of colostrum from PregSure vaccinated dams, the questionnaire covered a wide range of potential risk factors, including general management practices, colostrum and milk feeding practices, vaccine and medicine use, and BVD control measures (Table 1). Details of the clinical picture, such as of the number of calves affected by BNP in previous years, were requested. A copy of the questionnaire is provided in the supplementary material (S1 File). The questionnaire used in the case-control study consisted of two parts: The first part asked about herd-level management information (the focus of the current study) and the second part asked about the detailed history of affected calves and chosen control calves (the focus of the previously published study [24]. Data used in the present study were not analysed for the previous publication [24].

**Table 1:** Risk factor groups included in study questionnaire of multi-country BNP case-control study.

Risk group	Subjects
General farm management	Production type, number of cattle, changes to herd size, production, other species being held on the farm, rearing of young stock, purchase and selling
Colostrum and milk feeding of calves	Detailed information on colostrum and milk feeding of calves within the first two weeks of life
Vaccinations	Vaccinations done routinely on the farm, detailed for age groups of cattle
Treatments	Medical treatments applied routinely to cattle
BVD-related	BVDV history on the farm, BVDV vaccinations in detail

Because of the detailed information required from case farms on vaccinations and the management of the case and control calves, it was considered best for the veterinarian to complete the questionnaire with the farmer during a face-to-face interview in their local language. In France and Germany, the local veterinarian was sent a copy of the translated questionnaire and was asked to complete this with the farmer and return it to the project coordinators in their respective country. After the questionnaires were obtained by the coordinators, the answers were checked for plausibility and completeness and, if necessary, veterinarians were contacted for clarification. In the Netherlands and Belgium, one project veterinarian visited all case farms and completed the questionnaire together with the farmer. Each case farm interview lasted 1.5 to 2 hours.

For control farms, less detailed information was required as there were no affected calves. Thus these farmers were contacted by telephone and a time for a telephone interview was arranged. Telephone interviews were carried out by the project coordinators in the respective countries.

All data were entered into a centralised database via a web-based data-entry form developed at Ghent University, Department of Reproduction, Obstetrics and Herd Health, using the open source software LimeSurvey (http://www.limesurvey.org). Data were then exported first to a Microsoft Excel spreadsheet for preliminary cleaning and then into the SAS for Windows 9.3 statistical software for coding, further cleaning and analysis.

### Data analysis

The database table consisted of 1462 records. If case farms had several BNP calves the farm was represented multiple times in the database table. Since for the present analysis only farm information was relevant, therefore duplicate records, representing information on different calves from the same farm, were deleted. If conflicting information had been entered for a farm, paper records were consulted for clarification. Records of case farms without a matched control farm and records with a high percentage of missing values were deleted from the analysis dataset. This resulted in a subset of data for analysis comprising 1250 observations.

Categorical variables with small numbers of observations in some categories were aggregated into a smaller number of categories based on epidemiologically and biologically sensible criteria. For the variable ‘Feeding colostrum from the calf’s own dam’ the category ‘always’ was compared with the combined responses ‘sometimes’ and ‘never’. For the other variables, the responses ‘always’ and ‘sometimes’ were combined and compared with ‘never’. Continuous variables were assessed for normality using quantile-quantile plots. They were also inspected for linearity using scatterplots and in the case of a non-linear relationship between the predictor variable and the case/control status, the predictor variable was categorised using quartiles.

Variables were categorised into five different risk factor groups, representing variables about general herd management, colostrum and milk feeding variables, vaccinations, treatments and BVD-related variables (Table 1).

The following variables were created based on information from the original questionnaire responses: vaccination on farm (i.e. whether any age-group was vaccinated), medication used on farm (whether any age-group was treated), BVDV-vaccine used (whether any BVDV vaccine had been used) and variables for each brand of BVDV-vaccine, which were mentioned in the questionnaires (PregSure, Bovilis BVD (MSD Animal Health), Bovidec BVD (Novartis Animal Health), Mucosiffa/Vacoviron (Merial), Mucobovin (Merial) and Rispoval-BVD (Pfizer Animal Health) [whether this brand had been used on any age-group at any time, either currently or in the past]). Additionally, the variable ‘BVD vaccination’ was created to distinguish between farms that had used PregSure (and possibly other BVDV vaccines) at any time in the past, those that had only used other BVDV vaccines and farms that had not used any BVDV vaccines. The variables based on the questions ‘was the herd BVD free?’ and ‘on what basis was BVD freedom determined?’ were not deemed to be risk factors for the occurrence of BNP and were therefore omitted from the analysis.

The analysis was conducted in three steps: first a descriptive analysis; second, a univariable analysis to assess the statistical significance of relationships between each putative risk factors and the occurrence of BNP and third, a multivariable analysis to identify the most important risk factors, controlling for confounding and testing biologically plausible interactions. In the univariable analysis, to assess the effect of matching, an unmatched and a matched analysis was conducted and results compared between the two. The unmatched analysis was performed using Mann-Whitney U tests for continuous variables and for categorical variables odds ratios were estimated using cross-tabulations with statistical significance determined using chi-squared tests. The matched analysis used conditional logistic regression analysis to estimate odds ratios for each variable with statistical significance being assessed using the Wald statistic. The crude odds ratios obtained in the unmatched analysis were mostly closer to 1 than those obtained in the conditional logistic regression analysis, indicating that matching had a positive effect [25]. The multivariable analysis was also conducted in two steps. The first step involved separate multivariable analyses for the five different risk factor groups (see Table 1). Variables that were statistically significant in each of the individual risk-factor group analyses were then included in the second step in an overall multivariable analysis. The multivariable analysis within each of the risk-factor groups included all variables within the group that had univariable p-values of < 0.20. If a variable was close to uniformity it was omitted from the multivariable analysis. Conditional logistic regression was used with backward stepwise variable selection based on the likelihood-ratio statistic with a p-value of < 0.05 for entry and > 0.1 for removal. To assess collinearity between variables, pair-wise associations were examined using polychoric correlations. If two variables were highly correlated (r > 0.8) only one was offered to the modelling algorithm, chosen based on biological reasoning or a lower p-value.

For the final model of the analysis across risk factor groups, first-order-interactions between the significant main effect variables were assessed for their statistical significance. Results are presented as odds ratios together with their 95% confidence intervals. All analyses were conducted using SAS for Windows version 9.3 (SAS Institute, Cary, North Carolina, USA).

## Results

### Descriptive results

In total, 212 farm records had to be omitted from the analysis due to duplication, incomplete data or not being matched with a case or control farm. Thus, 1250 farm records remained in the final database, of which 363 were case farms and 887 were control farms. Belgium contributed 70 case and 160 control farms, France 116 cases and 297 controls, Germany 77 cases and 203 controls and The Netherlands 100 cases and 227 controls. In total, there were 812 dairy farms, 228 beef farms and 210 mixed farms. These were not evenly distributed between the countries (Table 2).

**Table 2:** Production type of farms included in multi-country BNP case control study.

Country	Case-control status	Dairy farms	Beef farms	Mixed farms
Belgium	Case	33	17	20
	Control	74	54	32
France	Case	48	36	32
	Control	139	110	48
Germany	Case	63	2	12
	Control	136	9	58
The Netherlands	Case	96	0	4
	Control	223	0	4

The types of BVD vaccine used on case and control farms are shown in Table 3. More than half of the control farms had not used any BVD vaccine, compared with only 7% of the case farms. More than 85% of the case farms had used the BVD vaccine PregSure, compared with 22% of the control farms. Also 22% of the control farms only used other BVD vaccines, i.e. not PregSure, compared with only 5% of the case farms.

**Table 3:** Distribution of case and control farms using different BVDV vaccines in multi-country BNP study (n = 1165).

BVD vaccination	Case farms (n = 351)	Control farms (n = 814)
No BVD vaccination	25 (7.1%)	454 (55.8%)
PregSure only	90 (25.6%)	28 (3.4%)
PregSure together with other BVD vaccines	219 (62.4%)	149 (18.3%)
Only other BVD vaccines, but no PregSure	17 (4.8)	183 (22.5%)

### Univariable analysis

Results of univariable analyses for each of the different risk factor groups are presented in S1–S8 Tables. In the risk factor group ‘General farm management’ the total number of cattle on the farm differed significantly between case and control farms, with a median of 150 cattle on case farms and 130 cattle on control farms (p = 0.012; S1 Table). The number of lactating and dry cows being held on the farm differed between case and control farms (median on case farms 70 cows and median on control farms 60.5 cows, p = 0.010). In dairy farms the median 305 day lactation production was 8500 kg on case farms and 8300 kg on control farms (p<0.001). In the risk factor group ‘Colostrum and milk feeding’ (S2 Table) calves on control farms were more frequently allowed to suckle ad libitum at their first feeding than on case farms (79% vs. 61%; p = 0.034). There were several differences between case and control farms regarding the source of colostrum. Amongst case farms, a lower proportion of calves obtained always the colostrum from their own dams than on control farms (p = 0.007). Feeding pooled colostrum from dams of the own farm was sometimes practised on 15% of the case farms and only on 8% of the control farms (p<0.001). Also colostrum substitutes without IgG were used more frequently on case farms than on control farms, although their use was limited (3% vs. 1%; p = 0.005). After the colostrum phase, case farms more frequently used milk powder (51% vs. 44%; p = 0.015), while control farms more frequently used bulk milk (48% vs. 40%; p<0.001). In the risk factor group ‘Vaccinations’ (S3–S6 Tables) the variables were analysed individually for each age group as well as at the entire herd level. There were significant differences in the use of vaccinations against BVD, IBR and BTV for each of the age groups and at whole herd level, with case farms using these vaccinations more frequently than control farms (p<0.001 for BVD; p<0.006 for IBR; p<0.002 for BTV). At entire herd level, vaccination against BRSV was also used more frequently on case farms than control farms (p = 0.035). Vaccinations against Trichophyton species were used in a small percentage of farms, but significantly more often on case than control farms (p = 0.045). Different routine treatments (S7 Table) were also used slightly more frequently on case than control farms (sulfonamides: 8% vs. 4%, p = 0.014; vitamin E/selenium: 19% vs. 14%, p = 0.012 and pour-on insecticides: 58% vs. 52%, p = 0.023). Regarding the ‘BVDV-related’ risk factor group (S8 Table) several variables differed significantly between case and control farms. Case herds had been free of BVD in the previous 12 months less frequently than control herds (43% vs. 50%; p = 0.031). A higher proportion of case farms had previously identified a persistently infected BVD animal (40% vs. 21%; p<0.001), and case farms more often had a BVD vaccination programme in place than on control farms (82% vs. 41%; p<0.001). On case farms, the most frequently cited reason for starting these vaccination programmes was an existing BVD problem (60%), while only 34% of the control farms with a BVD vaccination programme cited this reason. At the time of the interview, 59% of case farms were still vaccinating against BVDV compared with only 33% of control farms (p<0.001). The case farms had more frequently used another BVD-vaccine in the past than the one they used at the time of the interview (84% vs. 33% of the control farms; p<0.001). Of the 337 (93%) case farms that had been using BVD vaccines, 92% used PregSure, 36% Bovilis, 10% Bovidec and 31% Rispoval BVD. Of the 416 (47%) control farms that had been using BVD vaccines, the corresponding percentages were 43%, 33%, 8% and 40%, respectively (p<0.001). Only 17% of the case farms used live attenuated BVD vaccines (Mucosiffa or Vaccoviron), compared with 26% of control farms (p = 0.005). Combining the use of the different vaccines into the variable ‘BVD vaccination’, only 7% of the case farms did not use any BVD vaccine, 88% used PregSure (with or without other BVD vaccines) and 5% used only vaccines other than PregSure. For the control farms, these percentages were 56%, 22% and 22% respectively (p<0.001).

### Multivariable analysis

Results of multivariable analyses for each of the five risk factor groups are shown in Table 4. From the risk factor group ‘General farm management’ the variables ‘increase in dairy herd size’ and ‘average 305 day lactation production’ only applied to dairy farms, while the variable ‘increase in beef herd size’ only applied to beef herds. A multivariable analysis excluding these variables resulted in the retention of only the variable ‘total number of cattle’ (p = 0.010; Table 4). From the risk factor group ‘Colostrum and milk feeding’, the variables ‘calves allowed to suckle ad lib’ and the percentages of calves receiving colostrum from their own dam and from different dams were excluded from multivariable analysis, as these parameters had a high percentage of missing values. Of the eight variables included in multivariable analysis, five variables remained in the final model of this risk factor group (colostrum from own dam, p = 0.010; pooled colostrum from same farm, p<0.001; colostrum substitute without IgG, p = 0,005; colostrum substitute with IgG, p = 0.039; bulk milk feeding, p<0.001; Table 4). In the risk factor group ‘Vaccination’ the herd-aggregated, and not the age-specific, variables were used because of strong correlations between specific vaccinations for the three age-groups. Only two variables remained in the final model for this risk factor group (vaccination against BVD, p<0.001 and vaccination against BTV, p = 0.025; Table 4). Three variables from the risk factor group ‘Treatments’ remained in the model (regular use of sulphonamides, p = 0.024, vitamin E/selenium, p = 0.027 and pour-on insecticides, p = 0.043; Table 4). Within the risk factor group ‘BVDV related variables’ several variables were correlated with each other. The variable ‘reason for starting a BVD vaccination programme’ was excluded from the multivariable analysis, as it would have limited the dataset to those farms that had a BVD vaccination programme in place. Only the variable ‘BVD vaccination’ was found to be significantly associated with the case-control status (Table 4).

**Table 4:** Results of multivariable conditional logistic regression analysis for each of five risk factor groups for a multi-country BNP case-control study.

Risk factor group (n)	Variable	Variable category	Adjusted odds ratio	95% confidence intervals	Wald test p value
General farm management (1239)	Total number of cattle		1.001	1.000–1.003	0.0099
Colostrum and milk feeding (1195)	Colostrum from own dam	Always	0.640	0.456–0.899	0.0101
		Sometimes / Never	1.000		
	Pooled colostrum from same farm	Always / Sometimes	3.035	1.885–4.885	<0.0001
		Never	1.000		
	Colostrum substitute without IgG	Always / Sometimes	6.597	1.761–24.705	0.0051
		Never	1.000		
	Colostrum substitute with IgG	Always / Sometimes	2.132	1.040–4.373	0.0388
		Never	1.000		
	Bulk milk feeding	Yes	0.550	0.397–0.761	0.0003
		No	1.000		
Vaccinations (1250)	BVD vaccination used by farm	Yes	4.619	3.313–6.439	<0.0001
		No	1.000		
	BTV vaccination used by farm	Yes	1.454	1.049–2.014	0.0245
		No	1.000		
Treatments (1250)	Sulfonamides used by farm	Yes	2.032	1.098–3.752	0.0240
		No	1.000		
	Suppl. Vitamin E and selenium	Yes	1.520	1.048–2.204	0.0273
		No	1.000		
	Insect repellent pour on used by farm	Yes	1.336	1.009–1.769	0.0433
		No			
BVDV related (1087)	BVD vaccination	No BVD vaccination	1.000		<0.0001
		PregSure used^a^	110.379	38.054–264.784	
		Other BVD vaccines used^b^	1.947	0.826–4.591	

^a^ PregSure vaccine used alone or other BVD vaccines as well

^b^ Other BVD-vaccines used, not PregSure

All variables retained in the individual risk factor group models were examined for correlations and then included in the combined multivariable analysis. A polychoric correlation coefficient of > 0.8 was found between the variable ‘BVD vaccination’ (BVD vaccine used, PregSure used, other BVD vaccine used) from the risk factor group ‘BVD related variables’ and ‘BVD vaccination used on farm’ (yes/no) from the risk factor group ‘Vaccinations’. The latter variable was omitted, because it contained less information than the former. The results for the final model are shown in Table 5. Three variables were retained: BVD vaccination, the use of colostrum from the calf’s own dam (always versus sometimes/never), and the use of pooled colostrum from the same farm (sometimes versus never). The use of PregSure had the highest odds ratio. A case farm had 107 times the odds (95% CI: 41.06 to 280.13) of using PregSure vaccine (on its own or together with other BVD vaccines) rather than no BVD vaccination at all compared with a control farm, adjusting for colostrum feeding practices. Case farms had 1.98 times the odds (95% CI: 1.13–3.42) of always having used the colostrum from the calf’s dam rather than sometimes compared with controls, adjusting for the use of BVD vaccines and the use of pooled colostrum. Also, case farms had 4.1 times the odds (95% CI: 1.92–8.75) of sometimes having used pooled colostrum rather than never, compared with control farms, adjusting for the use of BVD vaccines and using colostrum from the own dam. No statistically significant two-way interactions were identified.

**Table 5:** Results of the final multivariable conditional model using the variables of the five individual risk groups within a multi-country BNP study (n = 1135).

Variable	Variable category	Adjusted odds ratio	95% confidence intervals	Wald test p value
BVD vaccination	No BVD vaccination	1.000		<0.0001
	PregSure used^a^	107.246	41.059–280.127	
	Other BVD vaccines used^b^	1.671	0.639–4.029	
Colostrum from own dam	Always used	1.976	1.131–3.421	0.0165
	Sometimes/Never used	1.000		
Pooled colostrum from own farm	Always / Sometimes used	4.095	1.918–8.746	0.0003
	Never	1.000		

^a^ PregSure vaccine used alone or other BVD vaccines as well

^b^ Other BVD-vaccines used, not PregSure

## Discussion

At the inception of this study, only limited information regarding the aetiology of BNP was available. It had been hypothesised that BVD vaccines in general, or one in particular (PregSure) could play an important role [17, 19, 26]. It had also been reported that the disease was associated with feeding colostrum from specific cows to newborn calves [1, 20]. To obtain an understanding of the underlying causal web, the current study investigated a wide variety of potential risk factors ranging from general herd management and vaccinations to feeding of animals, especially calves. By including cattle farms in four countries, a variety of husbandry and production systems were represented in the study. Therefore, the results obtained in the present study are not limited to one region or one specific management system, but instead apply across a range of systems. Furthermore, including four countries increased the likelihood of obtaining sufficient cases from different production systems, given that BNP is quite a rare disease. Prior to this study, only two smaller-scale case-control studies had been conducted [17, 19]. Both studies reported a statistically significant association between the use of PregSure and the occurrence of BNP. However, although PregSure had been used on thousands of cattle farms in Europe, only about 6000 BNP cases have been reported in Europe [10]. This provides strong indications that in addition to PregSure vaccination other component causes must be required which together form a sufficient cause that will result in BNP. Farm characteristics and herd management factors had to be considered amongst these, but the studies conducted so far had insufficient statistical power and represented insufficient variation in production systems to exclude the existence of such component causes. The current study was designed to fulfil that knowledge gap. It was not possible to recruit the target number of 100 case farms and up to 400 control farms for each country during the one-year study period due to limited case reporting in some countries. But the study still generated a database with 363 case and 887 control farms suitable for analysis, thereby providing sufficient statistical power to detect smaller effect sizes and representing a broader spectrum of cattle farming systems than any of the previous studies.

The case definitions used in each of the countries before the beginning of the study differed slightly with regard to blood value thresholds or pathological findings. For the present study, we used the same criteria across the four study countries. A calf was only classified as a BNP case if it showed clinical signs and if panmyelophthisis was found in bone marrow histology and/or if thrombocytopenia (values below 150 x 109/litre) and leukocytopenia (values below <5 x 109/litre) were present. Pardon et al. [21] used different cut-off values of <100 x 109/litre thrombocytes and <3 x 109/litre leucocytes, while Friedrich et al. [12] used values of < 200 x 109/litre thrombocytes and <4 x 109/litre leucocytes. The cut-off values chosen in the present study were more stringent than the officially used ones, which varied across the study countries. Therefore, we are confident that case farms did not include any false positives. Recruitment of control farms was based on the absence of clinical cases according to veterinarians’ and farmers’ recollection. We cannot completely exclude the possibility that some of the control farms were false negatives. If a significant number of control farms had been misclassified this would have resulted in the effect sizes of the risk factors to have been underestimated. But this is unlikely to have been the case due to the pathognomonic clinical signs of BNP. We took particular care to ensure that control farms were highly likely to be unaffected: first the local veterinarians had to confirm that they had never seen cases on these farms and second the farm managers had to confirm that they had not had calves with clinical signs in the past. Thus, if some control farms were still misclassified, it at most would be a very small percentage of control farms and therefore unlikely to result in any meaningful bias of the effect estimates.

The possibility of bias resulting from different approaches to questionnaire administration on case farms in the four countries cannot be excluded. In the Netherlands and Belgium, one project veterinarian visited each of the case farms, maximising consistency of data collection. In Germany and France, local veterinarians visited the case farms, hence potentially introducing potential bias as different individuals were involved in questionnaire administration. Furthermore, the questionnaire was extensive and focused on different themes such as general farm management, vaccinations and milk feeding of calves. We attempted to reduce bias by phrasing very precise questions, and whenever possible using records kept by farmers and veterinarians and by checking for potential errors and misinterpretations once the questionnaires had been returned to the country project centres. If there were any indications of such errors, the local veterinarian and/or farmer were phoned for clarification. Furthermore, some questions were repeated in different ways in different sections of the questionnaire to provide an internal cross-check and to help minimise recall bias. Additionally, farm managers of case farms were interviewed within a period of 2–10 weeks of the case being diagnosed to further minimise recall bias. Data collection on control farms offered less potential for heterogeneity in information bias between countries, as all interviews were conducted by telephone in all four countries. It is possible that face-to-face interviews have resulted in better data quality than telephone interviews, thereby causing differential information bias between case and control farms. However, as the questions used for comparison between case and control farms were all about general herd and calf management, this bias was considered negligible.

Further actions for reducing potential bias were taken at the analysis level. Data collected on BVDV related issues were cross-checked and combined into a reduced set of new variables, thus providing more reliable information and resulting in less multicollinearity between the variables. Data collected for different age groups were analysed separately as well as aggregated at the whole herd level, as the answers were highly collinear between the age groups.

In the final conditional logistic regression model, only three variables were significant: BVDV vaccination, feeding of colostrum from calf’s own dam, and feeding of pooled colostrum. The variable ‘BVD vaccination’ incorporated vaccination information for all age groups and also the type of vaccine used (no BVD vaccination, PregSure used with or without other BVD vaccines, and other BVD vaccines used except PregSure). This variable had the largest effect size in the multivariable analysis. Of all the risk factors, the highest odds ratio was for using PregSure vaccine either alone or in combination with other BVDV vaccines. The association between the use of PregSure and case-control status is consistent with results of previous case-control studies, which reported odds ratios of 40.8 and 1292 for the use of PregSure [17, 19] and of other studies that examined the pathogenesis of BNP [13, 21–23, 27–30]. This factor was also found to be significantly associated with the occurrence of BNP in our analysis at animal level [24]. This finding is supported by the absence of BNP reports from Switzerland, Denmark and Austria, where no BVD vaccination is allowed.

Another risk factor retained in the final multivariable model was the use of colostrum from the calf’s own dam. The involvement of colostrum in the development of BNP has already been confirmed in experimental studies [1, 20]. In our study, case farms had higher odds than control farms of always (rather than only sometimes) using colostrum from the calf’s own dam. Colostrum is important for a calf’s health and development [31–33] and it is generally recommended to use colostrum from the dam or from other dams on the same farm, in order to confer farm-specific immunity [34]. Most farmers in this study used colostrum from the calf’s own dam. If these cows had previously had a BNP-affected calf, then continuing to feed this specific colostrum, even from subsequent lactations, posed a risk of further occurrences of BNP, due to the potentially still occurring production of the specific antibodies.

The third risk factor was the use of pooled colostrum. Case farms had higher odds than control farms of using colostrum pooled from multiple cows from their own farm. This finding is supported by experimental studies, in which calves were more frequently and more severely affected if given pooled colostrum from BNP cows rather than colostrum from one individual cow which previously had at least one BNP calf [35, 36]. In previous farm-level case-control studies for BNP this variable was either not included in questionnaires [19, 26] or was statistically also significant [17]. In our previously published study about calf-level factors associated with BNP, pooled colostrum was also found to be a risk factor for the occurrence of BNP [24]. The likely reason for an increased risk associated with using pooled colostrum is the mixing of different alloantibody specificities included in the colostra of different cows and thus the increased risk of including at least one colostrum source with antibodies in it that are able to result BNP in the calf [35, 36].

Given the study had sufficient statistical power to identify risk factors with relatively small odds ratios (OR ≥1.6) and it representing a variety of cattle production systems in European countries, it can be concluded with some confidence that farm characteristics and herd management factors, other than PregSure vaccination of the dam and colostrum usage, are unlikely to have an important role in the causation of BNP. This leads to the conclusion that factors other than those investigated are component causes that together with PregSure vaccination of the dam and colostrum feeding form a sufficient cause for the occurrence of BNP in a calf. Considering the very low incidence of the disease despite widespread use of PregSure [23], it is likely that genetic factors are important. Given that the study included farms from four different countries with a variety of breeds, the genetic predisposition of cows and/or calves must occur across different cattle breeds. Different researchers have suggested that the expression of MHC class I [29, 30] or other candidate proteins [37, 38] may play a role in BNP pathogenesis.

This current study conducted at herd level provides further evidence for a strong association at farm level between the occurrence of BNP and the use of PregSure vaccine in cows, as well as of the risk associated with either feeding colostrum from the calf’s dam or of pooled colostrum. It can also be concluded with a fairly high degree of confidence that no other farm characteristic or herd management factor studies here are risk factors for BNP. But given that BNP incidence has been very low in the study countries, despite the two identified exposures applying to very large numbers of calves in Europe, other important component causes which together represent a sufficient cause of BNP still need to be identified. The genetic predisposition of the dams and/or calves is likely to be one such factor [39].

## Supporting information

S1 File Questionnaire used in the multi-county BNP study. (DOCX)

S1 Table Results of the univariable conditional logistic regression analysis–Risk factor group ‘General farm management’. Statistically significant parameters (p ≤ 0.05) are indicated in bold.
(DOC)

S2 Table Results of the univariable conditional logistic regression analysis–Risk factor group ‘Colostrum and milk feeding’. Statistically significant parameters (p ≤ 0.05) are indicated in bold.
(DOC)

S3 Table —Results of the univariable conditional logistic regression analysis–Risk factor group ‘Vaccination’ in young stock. Statistically significant parameters (p ≤ 0.05) are indicated in bold.
(DOC)

S4 Table Results of the univariable conditional logistic regression analysis–Risk factor group ‘Vaccination’ in breeding heifers. Statistically significant parameters (p ≤ 0.05) are indicated in bold.
(DOC)

S5 Table Results of the univariable conditional logistic regression analysis–Risk factor group ‘Vaccination’ in mature cows. Statistically significant parameters (p ≤ 0.05) are indicated in bold.
(DOC)

S6 Table Results of the univariable conditional logistic regression analysis–Risk factor group ‘Vaccination’ on farm-level. Statistically significant parameters (p ≤ 0.05) are indicated in bold.
(DOC)

S7 Table Results of the univariable conditional logistic regression analysis–Risk factor group ‘Treatment’. Statistically significant parameters (p ≤ 0.05) are indicated in bold.
(DOC)

S8 Table Results of the univariable conditional logistic regression analysis–Risk factor group ‘BVDV-related’. Statistically significant parameters (p ≤ 0.05) are indicated in bold.
(DOC)
