# Supplementary material for: Herd-level animal management factors associated with the occurrence of bovine neonatal pancytopenia in calves in a multi-country study
Source: PLoS One. 2017 Jul 5;12(7):e0179878. doi: 10.1371/journal.pone.0179878 (PMC5497972; doi:10.1371/journal.pone.0179878)
Supplement: S1 File — (DOCX) [file pone.0179878.s001.docx]

**Bovine neonatal pancytopenia questionnaire**

Questions refer to the previous 12 months except where specifically stated

1. Farm details

| Case no. |  | Control no. | |  | Linked to case no. | |  |
| --- | --- | --- | --- | --- | --- | --- | --- |
| Country | | |  | | | | |
| Owner | | |  | | | | |
| Farm Number , ID | | |  | | | | |
| Address | | |  | | | | |
| Telephone | | |  | | | | |
| Email (owner): | | |  | | | | |
| Veterinarian | | |  | | | | |
| Address | | |  | | | | |
| Telephone | | |  | | | | |
| Email (vet): | | |  | | | | |
| Production type | | |  | | | | |
| Total number of cattle at time of interview (incl. Young stock) | | | Dairy: | | | Beef: | |
| Herd size increase / decrease (delete as appropriate) in last 12 months | | | Dairy: increase/decrease | | | Beef: increase/decrease | |
| Number of lactating and dry cows | | | Dairy: | | | Beef: | |
| Dairy: average 305 day lactation production | | |  | | |  | |
| Other species present on the farm:  Sheep:  Goats: | | | *Yes/no if yes: number:*  *Yes/no if yes: number:* | | | | |
| Seasonal calving (at least 60% calving within 3 months) | | | *Yes/no if yes, in which months:* | | | | |
| Do you buy in dairy cows | | | *Yes/no* | | | | |
| Replacements heifers were   - Reared on own farm - Reared in a farm specialising in young stock rearing if so, please state ID/address - Purchased from other farms | | | *Yes/no Percent of replacement: _________*  *Yes/no Percent of replacement: _________*  *(Farm ID: _______________)*  *Yes/no Percent of replacement: _________* | | | | |
| Having sold calves less than 4 weeks of age?  If yes, what age were they normally sold? What proportion of heifer calves were sold?  What proportion of bull calves were sold? | | | *Yes/no*  *Proportion:*  *Proportion:* | | | | |

Calf and colostrum management (general management)

Definition of colostrum: obtained from the first two milkings after calving

| 1. Were calves allowed to suckle own dam?   Were calves able to suckle ad lib? | *Yes/no*  *Yes/no* |
| --- | --- |
| 1. Did you routinely drench the calves with colostrum? | *No / Yes, when the calves do not take offered colostrum / Yes, always*  *If yes: amount in litres:* |
| 1. Total amount of colostrum given to calf within first 12 hours? | *Liters if not suckling own dam:* |
| 1. Time from birth to when first colostrum was offered? | *< 1 hour; 1-2 hours; 2-6 hours; > 6 hours* |
| 1. How many times was colostrum offered to calves within first 24 hours on average? |  |
| 1. For how many days was colostrum given to calves on average?   For how many days was the calf allowed to suckle ad lib on average? |  |
| 1. Source of the colostrum:   own dam?  Different dam from same farm?  Different dam from different farm?  Pooled colostrum of own farm?  Pooled colostrum of different farm?  Colost. substitutes without IgG in it only?    Colost. substitutes with IgG in it only?    Colostrum substitutes additionally? | *(may select more than one)*  *Always / Sometimes: in... .....% calves/ Never*  *Always / Sometimes: in... .....% calves/ Never*  *Always / Sometimes: in... .....% calves/ Never*  *Always / Sometimes: in... .....% calves/ Never*  *Always / Sometimes: in... .....% calves/ Never*  *Always / Sometimes: in... .....% calves/ Never if yes, which brand?___________________*  *Always / Sometimes: in... .....% calves/ Never if yes, which brand?___________________*  *Always / Sometimes: in... .....% calves/ Never*  *if yes, which brand?___________________* |

**Milk feeding (please tick appropriate)**

| Milk powder, please state brand |  |
| --- | --- |
| Raw milk |  |
| Bulk milk |  |
| Milk from cows with high SCC or clinical mastitis |  |
| Withdrawn/discarded milk (e.g. from cows  treated for mastitis) |  |
| Other |  |

**Vaccinations within the last 12 months**

| Disease | Calves up to 6 months | Young stock (> 6 mo) | Breeding heifers | Mature cows | Product name, group of animals treated and remarks (if unknown please state ‘unknown’) |
| --- | --- | --- | --- | --- | --- |
| BVD | Yes/no | Yes/no | Yes/no | Yes/no |  |
| IBR | Yes/no | Yes/no | Yes/no | Yes/no |  |
| BTV | Yes/no | Yes/no | Yes/no | Yes/no |  |
| BRSV | Yes/no | Yes/no | Yes/no | Yes/no |  |
| Trichophyty | Yes/no | Yes/no | Yes/no | Yes/no |  |
| Lungworm | Yes/no | Yes/no | Yes/no | Yes/no |  |
| Rota/Corona | Yes/no | Yes/no | Yes/no | Yes/no |  |
| Pasteurella | Yes/no | Yes/no | Yes/no | Yes/no |  |
| PI3 | Yes/no | Yes/no | Yes/no | Yes/no |  |
| Lepto-spirosis | Yes/no | Yes/no | Yes/no | Yes/no |  |
| Others: please state: | Yes/no | Yes/no | Yes/no | Yes/no |  |

**Routine medication use within the last 12 months (medication that is given regularly to groups of animals or all animals in the herd)**The following section relates to routine treatments given to groups of animals.

| Product | Yes/no | Product names  (if unknown please state ‘unknown’) |
| --- | --- | --- |
| Sulfonamides |  |  |
| Vit E/Se |  |  |
| Standard antibiotic treatments |  |  |
| Insecticides on calves  Eartags  Pour on |  |  |
| Insecticides on cows  Eartags  Pour on |  |  |
| Antiparasitic treatments |  |  |
| Others |  |  |

**Bovine viral diarrhea (BVD)**

| Was the herd BVD free during the last 12 months? | YES / NO / unknown | (indicate date since when BVD free: ) |
| --- | --- | --- |
| If BVD free, on what basis is this determined? | No clinical signs  Control programme – test and cull  Herd test  Other (describe): | |
| Have you had a confirmed BVD animal (PI) on your farm within the last 12 months? | YES / NO / Not monitoring for PIs |  |
| Have you had confirmed PI animals before the last 12 months (back to 2005)? | YES / NO / Not monitoring for PIs | If yes, which year? |
| Was there a BVD vaccination programme? | YES / NO | (indicate year that the programme started and the programme) |
| Reason for starting this vaccination:  Had a BVD problem on farm  To prevent the farm from having a  BVD problem  Unknown status  Others: ______________ | YES / NO  YES / NO  YES / NO  YES / NO |  |
| Currently still vaccinating against BVD? | Yes/no |  |
| Which BVDV vaccine is currently used? |  | |
| Have you used another BVDV vaccine in the past? | Which one? When did you change to the current vaccine? | |
| Last BVD vaccination date: |  | |
| Do you vaccinate according to the manufactures instructions?  e.g. if using PregSure BVD, for primary vaccination, do you vaccinate twice within 3 weeks followed by yearly boosters?  If you use Bovilis BVD do you vaccinate twice with an interval of 4 weeks? | Yes/no |  |
| Did you administer vaccine simultaneously with vaccines against other diseases?  If yes, which vaccines (and dates): | Yes/no |  |
