# Supplementary material for: Herd-level animal management factors associated with the occurrence of bovine neonatal pancytopenia in calves in a multi-country study
Source: PLoS One. 2017 Jul 5;12(7):e0179878. doi: 10.1371/journal.pone.0179878 (PMC5497972; doi:10.1371/journal.pone.0179878)
Supplement: S1 Table — Statistically significant parameters (p ≤ 0.05) are indicated in bold. (DOC) [file pone.0179878.s002.doc]

## Table S1 - Results of the univariable conditional logistic regression analysis – Risk factor group ‘General farm management’

Statistically significant parameters (p ≤ 0.05) are indicated in bold.

| **General farm management Variables** | **n** | **% missing** | **Variable category** | **No. cases (%)** | **No. controls**  **(%)** | **Cond. odds ratio** | **95% confidence interval** | **Wald test p value** |
| --- | --- | --- | --- | --- | --- | --- | --- | --- |
| Production type | 1240 | 0 | Dairy | 240 (66) | 572 (64) | 1 |  | 0.0669 |
|  |  |  | Beef | 55 (15) | 173 (20) | 0.412 | 0.174 – 0.977 |  |
|  |  |  | Mixed | 68 (19) | 142 (16) | 1.097 | 0.714 – 1.687 |  |
| **Total number of cattle** | **1240** | **0** | **Median** | **150** | **130** | **1.001** | **1.000-1.003** | **0.0120** |
|  |  |  | **1^st^ – 3^rd^ quartile** | **100-215** | **90-194** |  |  |  |
| Dairy herd size | 991 | 21 | Same | 196 (68) | 438 (62) | 1 |  | 0.1406 |
|  |  |  | Decrease | 20 (7) | 41 (6) | 1.206 | 0.650 – 2.236 |  |
|  |  |  | Increase | 74 (25) | 222 (32) | 0.735 | 0.532 – 1.033 |  |
| Beef herd size | 291 | 69 | Same | 72 (67) | 218 (77) | 1 |  | 0.4382 |
|  |  |  | Decrease | 8 (8) | 22 (8) | 1.177 | 0.455 – 3.039 |  |
|  |  |  | Increase | 27 (25) | 44 (15) | 1.552 | 0.793 – 3.049 |  |
| **Number of lactating and dry cows** | **1140** | **9** | **Median** | **70** | **60.5** | **1.005** | **1.001 – 1.008** | **0.0099** |
|  |  |  | **1^st^ – 3^rd^ quartile** | **50-100** | **41-85** |  |  |  |
| **Dairy: avg. 305 day lactation production (in 1000 kg)** | **955** | **27** | **Median** | **8.5** | **8.3** | **1.329** | **1.137 – 1.554** | **0.0004** |
|  |  |  | **1^st^ – 3^rd^ quartile** | **7.9-9.5** | **7.5-9.0** |  |  |  |
| Sheep present on farm | 1245 | 0.4 | Yes | 26 (7) | 73 (8) | 0.923 | 0.560 – 1.521 | 0.7526 |
|  |  |  | No | 336 (93) | 810 (92) | 1.000 |  |  |
| Goats present on farm | 1244 | 0.5 | Yes | 13 (4) | 41 (5) | 0.793 | 0.402 – 1.567 | 0.5047 |
|  |  |  | No | 384 (96) | 842 (95) | 1.000 |  |  |
| Seasonal calving | 1239 | 0.9 | Yes | 47 (13) | 151 (17) | 0.654 | 0.416 – 1.026 | 0.0648 |
|  |  |  | No | 312 (87) | 729 (83) | 1.000 |  |  |
| Buying in cows | 1090 | 13 | Yes | 56 (17) | 36 (18) | 0 949 | 0 665 – 1.355 | 0.7728 |
|  |  |  | No | 267 (83) | 732 (82) | 1.000 |  |  |
| Replacement heifers reared only on own farm | 1250 | 0 | Yes | 313 (86) | 772 (87) | 1.035 | 0.710 – 1.510 | 0.8567 |
|  |  |  | No | 50 (14) | 115 (13) | 1.000 |  |  |
| Selling calves less than 4 weeks of age | 1231 | 2 | Yes | 242 (67) | 541 (62) | 1.267 | 0.857 – 1.872 | 0.2355 |
|  |  |  | No | 120 (33) | 328 (38) | 1.000 |  |  |
| Age of calves sold | 648 | 48 | Median | 15 | 15 | 0.957 | 0.866 – 1.058 | 0.3890 |
|  |  |  | 1^st^ – 3^rd^ quartile | 14-15 | 14-15 |  |  |  |
