# Supplementary material for: Herd-level animal management factors associated with the occurrence of bovine neonatal pancytopenia in calves in a multi-country study
Source: PLoS One. 2017 Jul 5;12(7):e0179878. doi: 10.1371/journal.pone.0179878 (PMC5497972; doi:10.1371/journal.pone.0179878)
Supplement: S2 Table — Statistically significant parameters (p ≤ 0.05) are indicated in bold. (DOC) [file pone.0179878.s003.doc]

## Table S2 - Results of the univariable conditional logistic regression analysis – Risk factor group ‘Colostrum and milk feeding’

Statistically significant parameters (p ≤ 0.05) are indicated in bold.

| **Colostrum and milk feeding Variables** | **n** | **% missing** | **Variable category** | **No. cases (%)** | **No. controls**  **(%)** | **Cond. odds ratio** | **95% confidence interval** | **Wald test p value** |
| --- | --- | --- | --- | --- | --- | --- | --- | --- |
| Calves allowed to suckle own dam | 1245 | 0.5 | Yes | 136 (37) | 340 (39) | 1.111 | 0.815 – 1.515 | 0.5051 |
|  |  |  | No | 227 (63) | 542 (61) | 1.000 |  |  |
| **Calves allowed to suckle ad lib** | **217** | **82** | **Yes** | **39 (61)** | **121 (79)** | **0.325** | **0.115 – 0.917** | **0.0336** |
|  |  |  | **No** | **25 (39)** | **32 (21)** | **1.000** |  |  |
| Routinely drenching calves with colostrum | 1234 | 1 | Yes | 105 (29) | 304 (35) | 0.782 | 0.608 – 1.006 | 0.0557 |
|  |  |  | No | 252 (71) | 573 (65) | 1.000 |  |  |
| Amount of colostrum given within first 12 hours (in litres) | 1186 | 5 | Median | 4 | 4 | 1.033 | 0.932 – 1.145 | 0.5373 |
|  |  |  | 1^st^ – 3^rd^ quartile | 3-6 | 3-6 |  |  |  |
| Time from birth to first colostrum | 1243 | 0.6 | < 1 hour | 203 (56) | 493 (57) | 1 |  | 0.5731 |
|  |  |  | 1-2 hours | 77 (21) | 181 (20) | 0.909 | 0.640 – 1.292 |  |
|  |  |  | 2-6 hours | 72 (20) | 175 (20) | 0.988 | 0.680 – 1.435 |  |
|  |  |  | > 6 hours | 8 (2) | 34 (4) | 0.557 | 0.237 – 1.310 |  |
| How many times colostrum offered within 24 hours | 1003 | 20 | Median | 3 | 3 | 1.031 | 0.871 – 1.221 | 0.7209 |
|  |  |  | 1^st^ – 3^rd^ quartile | 2-3 | 2-3 |  |  |  |
| Days colostrum offered (censored at 5 days) | 1235 | 1 | Median | 1 | 2 | 0.994 | 0.695 – 1.422 | 0.9733 |
|  |  |  | 1^st^ – 3^rd^ quartile | 1-2 | 1-5 |  |  |  |
| *Colostrum from own dam* | 1240 | 0.8 | Always | 201 (56) | 447 (51) | 1.344 | 0.985 – 1.832 | 0.0620 |
|  |  |  | Some-times | 161 (44) | 426 (48) | (sometimes + never)  1.000 |  |  |
|  |  |  | Never | 0 | 5 (1) |  |  |  |
| **How many % of calves receive colostrum from own dam** | **580** | **54** | **Median** | **90%** | **95%** | **0.977** | **0.961 – 0.994** | **0.0068** |
|  |  |  | **1^st^ – 3^rd^ quartile** | **80-95** | **85-96** |  |  |  |
| Colostrum from different dams of the same farm | 1227 | 2 | Always | 6 (2) | 11 (1) | 1.105 always + sometimes | 0.820 – 1.488 | 0.5112 |
|  |  |  | Some-times | 248 (69) | 588 (68) |  |  |  |
|  |  |  | Never | 105 (29) | 269 (31) | 1.000 |  |  |
| How many % of calves receive colostrum from different dams (same farm*)* | 824 | 34 | Median | 10% | 5% | 1.011 | 0.998 – 1.024 | 0.0982 |
|  |  |  | 1^st^ – 3^rd^ quartile | 5-20 | 4-14 |  |  |  |
| Colostrum from different dams of different farms | 1224 | 2 | Always | 1 (0) | 5 (1) | 0.782 always + sometimes | 0.443 – 1.380 | 0.3961 |
|  |  |  | Some-times | 22 (6) | 60 (7) | 1.000 |  |  |
|  |  |  | Never | 334 (94) | 802 (92) |  |  |  |
| **Pooled colostrum from own farm** | **1223** | **2** | **Always** | **1 (0)** | **2 (0)** | **2.729** always + sometimes | **1.746 – 4.264** | **<0.0001** |
|  |  |  | **Some-times** | **52 (15)** | **68 (8)** |  |  |  |
|  |  |  | **Never** | **303 (85)** | **797 (92)** | **1.000** |  |  |
| Pooled colostrum from different farms | 1224 | 2 | Always | 1 (0) | 1 (0) | 0.337 always + sometimes | 0.041 – 2.754 | 0.3100 |
|  |  |  | Some-times | 1 (0) | 6 (1) |  |  |  |
|  |  |  | Never | 356 (99) | 859 (99) | 1.000 |  |  |
| **Colostrum substitute without IgG** | **1232** | **1** | **Always** | **2 (1)** | **1 (0)** | **4.693** always + sometimes | **1.617 – 13.619** | **0.0045** |
|  |  |  | **Some-times** | **11 (3)** | **10 (1)** |  |  |  |
|  |  |  | **Never** | **345 (96)** | **863 (99)** | **1.000** |  |  |
| Colostrum substitute with IgG | 1225 | 2 | Always | 2 (1) | 3 (0) | 1.886 always + sometimes | 0.991 – 3.590 | 0.0532 |
|  |  |  | Some-times | 19 (5) | 33 (4) |  |  |  |
|  |  |  | Never | 334 (94) | 834 (96) | 1.000 |  |  |
| Colostrum substitute given additionally to colostrum | 1222 | 2 | Always | 6 (2) | 15 (2) | 1.011 always + sometimes | 0.616 – 1.657 | 0.9664 |
|  |  |  | Some-times | 25 (7) | 62 (7) |  |  |  |
|  |  |  | Never | 324 (91) | 790 (91) | 1.000 |  |  |
| **Milk powder** | **1250** | **0** | **Yes** | **186 (51)** | **392 (44)** | **1.412** | **1.070 – 1.863** | **0.0147** |
|  |  |  | **No** | **177 (49)** | **495 (56)** | **1.000** |  |  |
| Raw milk | 1250 | 0 | Yes | 280 (77) | 722 (81) | 0.763 | 0.551 – 1.058 | 0.1047 |
|  |  |  | No | 83 (23) | 165 (19) | 1.000 |  |  |
| **Bulk milk** | **1250** | **0** | **Yes** | **145 (40)** | **428 (48)** | **0.543** | **0.401 – 0.737** | **<0.0001** |
|  |  |  | **No** | **218 (60)** | **469 (52)** | **1.000** |  |  |
| Milk from cows with high somatic cell count or clinical mastitis | 1250 | 0 | Yes | 144 (40) | 362 (41) | 0.881 | 0.662 – 1.172 | 0.3837 |
|  |  |  | No | 219 (60) | 525 (59) | 1.000 |  |  |
| Withdrawn/ discarded milk | 1250 | 0 | Yes | 136 (37) | 293 (33) | 1.200 | 0.898 – 1.602 | 0.2172 |
|  |  |  | No | 227 (63) | 594 (67) | 1.000 |  |  |
| Other milk fed | 1250 | 0 | Yes | 0 (0) | 17 (2) | Not defined |  | 0.9807 |
|  |  |  | No | 363 (100) | 870 (98) |  |  |  |

## 
