# Supplementary material for: Herd-level animal management factors associated with the occurrence of bovine neonatal pancytopenia in calves in a multi-country study
Source: PLoS One. 2017 Jul 5;12(7):e0179878. doi: 10.1371/journal.pone.0179878 (PMC5497972; doi:10.1371/journal.pone.0179878)
Supplement: S3 Table — Statistically significant parameters (p ≤ 0.05) are indicated in bold. (DOC) [file pone.0179878.s004.doc]

## Table S3 - Results of the univariable conditional logistic regression analysis – Risk factor group ‘Vaccination’ in young stock

Statistically significant parameters (p ≤ 0.05) are indicated in bold.

| **Vaccinations Variables** | **n** | **% missing** | **Variable category** | **No. cases (%)** | **No. controls**  **(%)** | **Cond. odds ratio** | **95% confidence interval** | **Wald test p value** |
| --- | --- | --- | --- | --- | --- | --- | --- | --- |
| **BVD** | **1250** | **0** | **Yes** | **162 (44)** | **205 (23)** | **3.206** | **2.328 – 4.416** | **<0.0001** |
|  |  |  | **No** | **201 (56)** | **682 (77)** | **1.000** |  |  |
| **IBR** | **1250** | **0** | **Yes** | **85 (23)** | **146 (16)** | **2.608** | **1.545 – 4.401** | **0.0003** |
|  |  |  | **No** | **278 (77)** | **741 (84)** | **1.000** |  |  |
| **BTV** | **1250** | **0** | **Yes** | **150 (41)** | **303 (34)** | **1.683** | **1.232 – 2.299** | **0.0011** |
|  |  |  | **No** | **213 (59)** | **584 (66)** | **1.000** |  |  |
| **BRSV** | **1250** | **0** | **Yes** | **159 (44)** | **341 (38)** | **1.374** | **1.023 – 1.845** | **0.0348** |
|  |  |  | **No** | **204 (56)** | **546 (62)** | **1.000** |  |  |
| **Trichophyty** | **1250** | **0** | **Yes** | **17 (5)** | **25 (3)** | **1.948** | **1.015 – 3.738** | **0.0449** |
|  |  |  | **No** | **346 (95)** | **862 (97)** | **1.000** |  |  |
| Lungworm | 1250 | 0 | Yes | 7 (2) | 12 (1) | 1.114 | 0.367 – 3.376 | 0.8492 |
|  |  |  | No | 356 (98) | 875 (99) | 1.000 |  |  |
| Rota/Corona | 1250 | 0 | Yes | 1 (0) | 7 (1) | 0.282 | 0.033 – 2.400 | 0.2465 |
|  |  |  | No | 362 (100) | 880 (99) | 1.000 |  |  |
| Pasteurella | 1250 | 0 | Yes | 44 (12) | 130 (15) | 0.782 | 0.508 – 1.203 | 0.2630 |
|  |  |  | No | 319 (88) | 757 (95) | 1.000 |  |  |
| Parainfluenza | 1250 | 0 | Yes | 132 (36) | 290 (33) | 1.219 | 0.897 – 1.655 | 0.2060 |
|  |  |  | No | 231 (64) | 597 (67) | 1.000 |  |  |
| Leptospriosis | 1250 | 0 | Yes | 0 | 0 |  |  | na |
|  |  |  | No | 363 (100) | 887 (100) |  |  |  |
| Others | 1250 | 0 | Yes | 23 (6) | 61 (7) | 0.924 | 0.502 – 1.702 | 0.8008 |
|  |  |  | No | 340 (94) | 826 (93) | 1.000 |  |  |
