# Supplementary material for: Herd-level animal management factors associated with the occurrence of bovine neonatal pancytopenia in calves in a multi-country study
Source: PLoS One. 2017 Jul 5;12(7):e0179878. doi: 10.1371/journal.pone.0179878 (PMC5497972; doi:10.1371/journal.pone.0179878)
Supplement: S4 Table — Statistically significant parameters (p ≤ 0.05) are indicated in bold. (DOC) [file pone.0179878.s005.doc]

## Table S4 - Results of the univariable conditional logistic regression analysis – Risk factor group ‘Vaccination’ in breeding heifers

Statistically significant parameters (p ≤ 0.05) are indicated in bold.

| **Vaccinations Variables** | **n** | **% missing** | **Variable category** | **No. cases (%)** | **No. controls**  **(%)** | **Cond. odds ratio** | **95% confidence interval** | **Wald test p value** |
| --- | --- | --- | --- | --- | --- | --- | --- | --- |
| **BVD** | **1250** | **0** | **Yes** | **203 (56)** | **236 (27)** | **4.416** | **3.217 – 6.060** | **<0.0001** |
|  |  |  | **No** | **160 (44)** | **651 (73)** | **1.000** |  |  |
| **IBR** | **1250** | **0** | **Yes** | **83 (23)** | **149 (17)** | **2.059** | **1.260 – 3.363** | **0.0039** |
|  |  |  | **No** | **280 (77)** | **738 (83)** | **1.000** |  |  |
| **BTV** | **1250** | **0** | **Yes** | **168 (46)** | **321 (36)** | **1.928** | **1.425 – 2.608** | **<0.0001** |
|  |  |  | **No** | **195 (54)** | **564 (64)** | **1.000** |  |  |
| BRSV | 1250 | 0 | Yes | 18 (5) | 32 (4) | 1.335 | 0.672 – 2.654 | 0.4095 |
|  |  |  | No | 345 (95) | 885 (96) | 1.000 |  |  |
| Trichophyty | 1250 | 0 | Yes | 1 (0) | 0 (0) | Not defined |  | 0.9713 |
|  |  |  | No | 363 (100) | 887 (100) |  |  |  |
| Lungworm | 1250 | 0 | Yes | 0 | 0 | Not defined |  | na |
|  |  |  | No | 363 (100) | 887 (100) |  |  |  |
| Rota/Corona | 1250 | 0 | Yes | 69 (19) | 152 (17) | 1.166 | 0.801 – 1.698 | 0.4225 |
|  |  |  | No | 294 (81) | 735 (83) | 1.000 |  |  |
| Pasteurella | 1250 | 0 | Yes | 3 (1) | 5 (1) | 1.108 | 0.201 – 6.115 | 0.9059 |
|  |  |  | No | 360 (99) | 882 (99) | 1.000 |  |  |
| Parainfluenza | 1250 | 0 | Yes | 8 (2) | 19 (2) | 0.779 | 0.301 – 2.017 | 0.6074 |
|  |  |  | No | 355 (98) | 868 (98) | 1.000 |  |  |
| Leptospriosis | 1250 | 0 | Yes | 0 | 0 | Not defined |  | na |
|  |  |  | No | 363 (100) | 887 (100) |  |  |  |
| Others | 1250 | 0 | Yes | 14 (4) | 69 (8) | 1.157 | 0.555 – 2.413 | 0.6974 |
|  |  |  | No | 349 (96) | 818 (92) | 1.000 |  |  |

## 
