# Supplementary material for: Herd-level animal management factors associated with the occurrence of bovine neonatal pancytopenia in calves in a multi-country study
Source: PLoS One. 2017 Jul 5;12(7):e0179878. doi: 10.1371/journal.pone.0179878 (PMC5497972; doi:10.1371/journal.pone.0179878)
Supplement: S5 Table — Statistically significant parameters (p ≤ 0.05) are indicated in bold. (DOC) [file pone.0179878.s006.doc]

## Table S5 - Results of the univariable conditional logistic regression analysis – Risk factor group ‘Vaccination’ in mature cows

Statistically significant parameters (p ≤ 0.05) are indicated in bold.

| **Vaccinations Variables** | **n** | **% missing** | **Variable category** | **No. cases (%)** | **No. controls**  **(%)** | **Cond. odds ratio** | **95% confidence interval** | **Wald test p value** |
| --- | --- | --- | --- | --- | --- | --- | --- | --- |
| **BVD** | **1250** | **0** | **Yes** | **193 (53)** | **228 (26)** | **4.315** | **3.128 – 5.954** | **<0.0001** |
|  |  |  | **No** | **270 (47)** | **659 (74)** | **1.000** |  |  |
| **IBR** | **1250** | **0** | **Yes** | **91 (25)** | **168 (19)** | **1.846** | **1.200 – 2.839** | **0.0053** |
|  |  |  | **No** | **272 (75)** | **719 (81)** | **1.000** |  |  |
| **BTV** | **1250** | **0** | **Yes** | **160 (44)** | **303 (34)** | **2.013** | **1.474 – 2.748** | **<0.0001** |
|  |  |  | **No** | **203 (56)** | **584 (66)** | **1.000** |  |  |
| BRSV | 1250 | 0 | Yes | 13 (4) | 22 (2) | 1.323 | 0.557 – 3.145 | 0.5262 |
|  |  |  | No | 350 (96) | 865 (98) | 1.000 |  |  |
| Trichophyty | 1250 | 0 | Yes | 4 (1) | 7 (1) | 2.000 | 0.528 – 7.574 | 0.3076 |
|  |  |  | No | 359 (99) | 880 (99) | 1.000 |  |  |
| Lungworm | 1250 | 0 | Yes | 2 (1%) | 0 (0%) | Not defined |  | 0.9745 |
|  |  |  | No | 361 (99) | 887 (100) |  |  |  |
| **Rota/Corona** | **1250** | **0** | **Yes** | **107 (29)** | **209 (24)** | **1.430** | **1.043 – 1.961** | **0.0263** |
|  |  |  | **No** | **256 (71)** | **678 (76)** | **1.000** |  |  |
| Pasteurella | 1250 | 0 | Yes | 1 (0) | 3 (0) | Not defined |  | 0.9821 |
|  |  |  | No | 362 (99) | 884 (100) |  |  |  |
| Parainfluenza | 1250 | 0 | Yes | 6 (2%) | 10 (1%) | 1.027 | 0.297 – 3.547 | 0.9662 |
|  |  |  | No | 357 (98) | 877 (99) | 1.000 |  |  |
| Leptospriosis | 1250 | 0 | Yes | 0 | 0 | Not defined |  | na |
|  |  |  | No | 363 (100) | 887 (100) |  |  |  |
| Others | 1250 | 0 | Yes | 14 (4) | 90 (10) | 1.363 | 0.658 – 2.822 | 0.4046 |
|  |  |  | No | 349 (96) | 797 (90) | 1.00 |  |  |

## 
