# Supplementary material for: Herd-level animal management factors associated with the occurrence of bovine neonatal pancytopenia in calves in a multi-country study
Source: PLoS One. 2017 Jul 5;12(7):e0179878. doi: 10.1371/journal.pone.0179878 (PMC5497972; doi:10.1371/journal.pone.0179878)
Supplement: S6 Table — Statistically significant parameters (p ≤ 0.05) are indicated in bold. (DOC) [file pone.0179878.s007.doc]

## Table S6 - Results of the univariable conditional logistic regression analysis – Risk factor group ‘Vaccination’ on farm level

Statistically significant parameters (p ≤ 0.05) are indicated in bold.

| **Vaccinations Variables** | **n** | **% missing** | **Variable category** | **No. cases (%)** | **No. controls**  **(%)** | **Cond. odds ratio** | **95% confidence interval** | **Wald test p value** |
| --- | --- | --- | --- | --- | --- | --- | --- | --- |
| **Farm-BVD** | **1250** | **0** | **Yes** | **231 (64)** | **305 (34)** | **4.814** | **3.459 – 6.699** | **<0.0001** |
|  |  |  | **No** | **132 (36)** | **582 (66)** | **1.000** |  |  |
| **Farm-IBR** | **1250** | **0** | **Yes** | **95 (26)** | **174 (20)** | **1.951** | **1.264 – 3.013** | **0.0026** |
|  |  |  | **No** | **268 (74)** | **713 (80)** | **1.000** |  |  |
| **Farm-BTV** | **1250** | **0** | **Yes** | **177 (49)** | **359 (40)** | **1.710** | **1.266 – 2.310** | **0.0005** |
|  |  |  | **No** | **186 (51)** | **528 (60)** | **1.000** |  |  |
| **Farm-BRSV** | **1250** | **0** | **Yes** | **164 (45)** | **344 (39)** | **1.442** | **1.074 – 1.936** | **0.0148** |
|  |  |  | **No** | **199 (55)** | **543 (61)** | **1.000** |  |  |
| **Farm-Trichophyty** | **1250** | **0** | **Yes** | **19 (5)** | **26 (3)** | **2.219** | **1.166 – 4.222** | **0.0152** |
|  |  |  | **No** | **344 (95)** | **861 (97)** | **1.000** |  |  |
| Farm-Lungworm | 1250 | 0 | Yes | 8 (2%) | 12 (1%) | 1.407 | 0.485 – 4.079 | 0.5297 |
|  |  |  | No | 355 (98) | 875 (99) | 1.000 |  |  |
| Farm-Rota/Corona | 1250 | 0 | Yes | 109 (30) | 221 (25) | 1.340 | 0.981 – 1.831 | 0.0662 |
|  |  |  | No | 254 (70) | 66 (75) | 1.000 |  |  |
| Farm-Pasteurella | 1250 | 0 | Yes | 46 (13) | 131 (15) | 0.801 | 0.522 – 1.229 | 0.3092 |
|  |  |  | No | 317 (83) | 756 (85) | 1.000 |  |  |
| Farm-Parainfluenza3 | 1250 | 0 | Yes | 133 (37) | 290 (33) | 1.219 | 0.897 – 1.655 | 0.2060 |
|  |  |  | No | 230 (63) | 597 (67) | 1.000 |  |  |
| Farm-Leptospirosis | 1250 | 0 | Yes | 0 (0) | 2 (0) | Not defined |  | 0.9794 |
|  |  |  | No | 363 (100) | 885 (100) |  |  |  |
| Farm-Other vaccinations | 1250 | 0 | Yes | 32 (9) | 89 (10) | 0.946 | 0.565 – 1.586 | 0.8341 |
|  |  |  | No | 331 (81) | 798 (90) | 1.000 |  |  |
