# Supplementary material for: Herd-level animal management factors associated with the occurrence of bovine neonatal pancytopenia in calves in a multi-country study
Source: PLoS One. 2017 Jul 5;12(7):e0179878. doi: 10.1371/journal.pone.0179878 (PMC5497972; doi:10.1371/journal.pone.0179878)
Supplement: S7 Table — Statistically significant parameters (p ≤ 0.05) are indicated in bold. (DOC) [file pone.0179878.s008.doc]

## Table S7 - Results of the univariable conditional logistic regression analysis – Risk factor group ‘Treatment’ on farm-level.

Statistically significant parameters (p ≤ 0.05) are indicated in bold.

| **Treatments Variables** | **n** | **% missing** | **Variable category** | **No. cases (%)** | **No. controls**  **(%)** | **Cond. odds ratio** | **95% confidence interval** | **Wald test p value** |
| --- | --- | --- | --- | --- | --- | --- | --- | --- |
| **Sulfonamides** | **1250** | **0** | **Yes** | **29 (8)** | **37 (4)** | **2.145** | **1.168 – 3.940** | **0.0139** |
|  |  |  | **No** | **334 (92)** | **850 (96)** | **1.000** |  |  |
| **VitE/Selenium** | **1250** | **0** | **Yes** | **69 (19)** | **126 (14)** | **1.604** | **1.111 – 2.316** | **0.0116** |
|  |  |  | **No** | **294 (81)** | **761 (86)** | **1.000** |  |  |
| Antiparasitica | 1250 | 0 | Yes | 234 (64) | 573 (65) | 0.910 | 0.650 – 1.273 | 0.5812 |
|  |  |  | No | 129 (36) | 314 (35) | 1.000 |  |  |
| Standard-Antibiotics | 1250 | 0 | Yes | 126 (35) | 267 (30) | 1.304 | 0.896 – 1.896 | 0.1655 |
|  |  |  | No | 237 (65) | 620 (70) | 1.000 |  |  |
| Insecticides Ear | 1250 | 0 | Yes | 36 (10) | 62 (7) | 1.392 | 0.863 – 2.245 | 0.1756 |
|  |  |  | No | 327 (90) | 825 (93) | 1.000 |  |  |
| **Pour on Insecticides** | **1250** | **0** | **Yes** | **212 (58)** | **464 (52)** | **1.368** | **1.035 – 1.808** | **0.0278** |
|  |  |  | **No** | **151 (42)** | **423 (48)** | **1.000** |  |  |
| other treatment | 1250 | 0 | Yes | 26 (7) | 77 (9) | 0.859 | 0.523 – 1.412 | 0.5489 |
|  |  |  | No | 337 (93) | 810 (91) | 1.000 |  |  |

## 
