# Supplementary material for: Herd-level animal management factors associated with the occurrence of bovine neonatal pancytopenia in calves in a multi-country study
Source: PLoS One. 2017 Jul 5;12(7):e0179878. doi: 10.1371/journal.pone.0179878 (PMC5497972; doi:10.1371/journal.pone.0179878)
Supplement: S8 Table — Statistically significant parameters (p ≤ 0.05) are indicated in bold. (DOC) [file pone.0179878.s009.doc]

## Table S8 - Results of the univariable conditional logistic regression analysis – Risk factor group ‘BVDV-related’

Statistically significant parameters (p ≤ 0.05) are indicated in bold.

| **BVD related Variables** | **n** | **% missing** | **Variable category** | **No. cases (%)** | **No. controls**  **(%)** | **Cond. odds ratio** | **95% confidence interval** | **Wald test p value** |
| --- | --- | --- | --- | --- | --- | --- | --- | --- |
| **Herd BVD free during last 12 months** | **1051** | **16** | **No** | **71 (20)** | **128 (14)** | **1.585** | **1.088 – 2.311** | **0.0309** |
|  |  |  | **Yes** | **158 (43)** | **441 (50)** | **1.000** |  |  |
|  |  |  | **Unknown** | **134 (37)** | **318 (36)** | **1.384** | **0.996 – 1.923** |  |
| Confirmed BVD PI animal within last 12 months | 1048 | 0 | No | 210 (58) | 543 (61) | 0.947 | 0.537 – 1.667 | 0.7842 |
|  |  |  | Yes | 21 (6) | 51 (6) | 1.000 |  |  |
|  |  |  | Not monitored | 131 (36) | 292 (33) | 1.115 | 0.790 – 1.574 |  |
| **Confirmed BVD PI animal before last 12 months** | **1250** | **0** | **No** | **102 (28)** | **350 (39)** | **1.000** |  | **<0.0001** |
|  |  |  | **Yes** | **146 (40)** | **187 (21)** | **2.955** | **2.069 – 4.220** |  |
|  |  |  | **Not monitored** | **115 (32)** | **350 (39)** | **1.125** | **0.789 – 1.605** |  |
| **BVD vaccination programme in place** | **1206** | **4** | **Yes** | **293 (82)** | **352 (41)** | **10.308** | **6.845 – 15.523** | **<0.0001** |
|  |  |  | **No** | **63 (18)** | **498 (59)** | **1.000** |  |  |
| **Reason for starting vaccination** | **828** | **34** | **BVD problem** | **194 (60)** | **173 (34)** | **13.555** | **3.953 – 46.478** | **<0.0001** |
|  |  |  | **Prevent BVD** | **84 (26)** | **107 (21)** | **12.011** | **3.384 – 42.628** |  |
|  |  |  | **Unknown status** | **39 (12)** | **175 (34)** | **3.681** | **0.997 – 13.586** |  |
|  |  |  | **others** | **5 (2)** | **51 (10)** | **1.000** |  |  |
| **Currently still vaccinating against BVDV** | **1179** | **6** | **Yes** | **210 (58)** | **274 (33)** | **3.480** | **2.523 – 4.800** | **<0.0001** |
|  |  |  | **No** | **149 (42)** | **546 (67)** | **1.000** |  |  |
| **Different Vaccine used in the past** | **913** | **27** | **Yes** | **289 (84)** | **256 (33)** | **13.950** | **8.924 – 21.809** | **<0.0001** |
|  |  |  | **No** | **57 (16)** | **523 (67)** | **1.000** |  |  |
| **PregSure used** | **1195** | **4** | **Yes** | **309 (92)** | **177 (43)** | **75.163** | **33.375 – 169.271** | **<0.0001** |
|  |  |  | **No** | **28 (8)** | **239 (57)** | **1.000** |  |  |
| **Bovilis used** | **1195** | **4** | **Yes** | **122 (36)** | **139 (33)** | **3.392** | **2.344 – 4.909** | **<0.0001** |
|  |  |  | **No** | **215 (64)** | **277 (67)** | **1.000** |  |  |
| **Bovidec used** | **1195** | **4** | **Yes** | **33 (10)** | **35 (8)** | **4.173** | **2.004 – 8.689** | **0.0001** |
|  |  |  | **No** | **304 (90)** | **381 (92)** | **1.000** |  |  |
| **Mucosiffa/ Vacoviron** | **1195** | **4** | **Yes** | **56 (17)** | **108 (26)** | **2.122** | **1.250 -3.602** | **0.0053** |
|  |  |  | **No** | **281 (83)** | **308 (74)** | **1.000** |  |  |
| Mucobovin used | 1195 | 4 | Yes | 12 (4) | 17 (4) | 1.949 | 0.736 – 5.163 | 0.1795 |
|  |  |  | No | 325 (96) | 399 (96) | 1.000 |  |  |
| **Rispoval BVD used** | **1195** | **4** | **Yes** | **103 (31)** | **163 (39)** | **2.404** | **1.628 – 3.550** | **<0.0001** |
|  |  |  | **No** | **234 (69)** | **253 (61)** | **1.000** |  |  |
| Last BVD vaccination date | 627 | 50 |  |  |  |  |  | 0.9602 |
| Vaccinate according to manufacturer’s instructions | 568 | 55 | Yes | 279 (90) | 244 (94) | 0.845 | 0.334 – 2.134 | 0.7212 |
|  |  |  | No | 30 (10) | 15 (6) | 1.000 |  |  |
| Vaccine administered together with other vaccines | 719 | 42 | Yes | 63 (19) | 59 (18) | 1.469 | 0.863 – 2.499 | 0.1561 |
|  |  |  | No | 258 (81) | 319 (82) | 1.000 |  |  |
| **BVD vaccination** | **1165** | **7** | **No BVD vaccine** | **25 (7)** | **454 (56)** | **1.000** |  | **<0.0001** |
|  |  |  | **PregSure** | **309 (88)** | **177 (22)** | **110.902** | **42.630 – 288.517** |  |
|  |  |  | **Other BVD vaccines** | **17 (5)** | **183 (22)** | **1.946** | **0.850 – 4.456** |  |
| **BVD vaccine used** | **1250** | **0** | **Yes** | **337 (93)** | **416 (47)** | **19.415** | **11.617 – 32.447** | **<0.0001** |
|  |  |  | **No** | **26 (7)** | **471 (53)** | **1.000** |  |  |
